# Interleukin-1 receptor–associated kinase 4 (IRAK4) plays a dual role in myddosome formation and Toll-like receptor signaling

**DOI:** 10.1074/jbc.RA118.003314

**Published:** 2018-08-03

**Authors:** Dominic De Nardo, Katherine R. Balka, Yamel Cardona Gloria, Vikram R. Rao, Eicke Latz, Seth L. Masters

**Affiliations:** From the ‡ Inflammation Division, Walter and Eliza Hall Institute of Medical Research, 1G Royal Parade, Parkville, Victoria 3052, Australia,; the § Department of Medical Biology, University of Melbourne, Parkville 3010, Australia,; the ¶ Institute of Innate Immunity, University Hospital, University of Bonn, Sigmund Freud Strasse 25, 53127 Bonn, Germany,; the ‖ Inflammation and Immunology, Pfizer Inc., Cambridge, Massachusetts 02139,; the ** Department of Infectious Diseases and Immunology, University of Massachusetts Medical School, Worcester, Massachusetts 01605, and; the ‡‡ German Center for Neurodegenerative Diseases, Bonn 53175, Germany

**Keywords:** innate immunity, Toll-like receptor (TLR), inflammation, myeloid differentiation primary response gene (88) (MYD88), macrophage, interleukin-1 receptor-associated kinase, IRAK4, IRAK1, myddosome, scaffold protein, NF-kappaB

## Abstract

Toll-like receptors (TLRs) form part of the host innate immune system, in which they act as sensors of microbial and endogenous danger signals. Upon TLR activation, the intracellular Toll/interleukin-1 receptor domains of TLR dimers initiate oligomerization of a multiprotein signaling platform comprising myeloid differentiation primary response 88 (MyD88) and members of the interleukin-1 receptor–associated kinase (IRAK) family. Formation of this myddosome complex initiates signal transduction pathways, leading to the activation of transcription factors and the production of inflammatory cytokines. To date, little is known about the assembly and disassembly of the myddosome and about the mechanisms by which these complexes mediate multiple downstream signaling pathways. Here, we isolated myddosome complexes from whole-cell lysates of TLR-activated primary mouse macrophages and from IRAK reporter macrophages to examine the kinetics of myddosome assembly and disassembly. Using a selective inhibitor of IRAK4's kinase activity, we found that whereas TLR cytokine responses were ablated, myddosome formation was stabilized in the absence of IRAK4's kinase activity. Of note, IRAK4 inhibition had only a minimal effect on NF-κB and mitogen-activated protein kinase (MAPK) signaling. In summary, our results indicate that IRAK4 has a critical scaffold function in myddosome formation and that its kinase activity is dispensable for myddosome assembly and activation of the NF-κB and MAPK pathways but is essential for MyD88-dependent production of inflammatory cytokines. Our findings suggest that the scaffold function of IRAK4 may be an attractive target for treating inflammatory and autoimmune diseases.

## Introduction

Families of highly conserved pattern recognition receptors expressed primarily by specialized immune cells, such as macrophages and dendritic cells, have evolved to recognize microbial and endogenous danger signals to activate an innate immune response (1). This response mediates a rapid and controlled acute inflammatory environment aimed at eliminating invading microorganisms via production of inflammatory mediators such as cytokines, chemokines, and interferons (2). The Toll-like receptors (TLRs)^4^ are an important family of pattern recognition receptors comprising 10 TLRs in humans and 12 expressed in mice (3). Whereas TLRs expressed on the cell surface typically recognize outer membrane components of bacteria, intracellular TLRs localized within acidified endolysosomal compartments detect microbial nucleic acids. TLRs exhibit a three-domain structure comprising a ligand recognition domain consisting of folded leucine-rich repeats at the N terminus, a central transmembrane-anchoring region, and a C-terminal cytoplasmic Toll/interleukin-1 (IL-1) receptor (TIR) domain that enables coupling of the TLRs to cytosolic TIR domain–containing adaptor proteins for triggering downstream signal transduction (4). Upon activation, TLRs initiate two main pathways that are predominantly dependent on the adaptors, myeloid differentiation primary response 88 (MyD88) and TIR domain–containing adapter–inducing interferon-β (TRIF). MyD88-dependent signal transduction is initiated through formation of a large oligomeric signaling complex containing molecules of MyD88 and members of the interleukin-1 receptor–associated kinase (IRAK) family, termed the “myddosome” (5–7). Interactions of MyD88 with the IRAKs are formed via common death domains (DDs), whereas the effector functions of the IRAKs are mediated via their central kinase domains. Myddosome formation promotes IRAK4 autoactivation, which initially activates IRAK1 and, at later time points, IRAK2 (8, 9). Putative binding motifs within IRAK1 and IRAK2 allow for transient recruitment of the E3 ubiquitin (Ub) ligase, TNF receptor–associated factor 6 (TRAF6), to the receptor complex to mediate its activation (10). Once activated, TRAF6 is released into the cytosol, where it triggers the IKK complex, leading to activation and nuclear translocation of NF-κB (11). In parallel, TRAF6 signaling also activates IRF5 and triggers the MAPK pathway to activate AP-1 and cAMP-response element–binding protein (12). The activation of these transcription factors culminates in production of potent pro-inflammatory cytokines and chemokines that elicit an acute inflammatory response. Recently, Bryant and colleagues (13) revealed that the kinetics of myddosome assembly correlated with NF-κB translocation and subsequent levels of TNF cytokine produced.

IRAK4 is critical for transducing positive TLR responses in both human and murine cells (14–16). Similarly, reconstitution of *Irak4*^−/−^ mice and murine cells with a kinase-dead version of IRAK4 was unable to rescue the *Irak4*^−/−^ phenotype, demonstrating that in mice, IRAK4 kinase activity is also vital (17–19). Interestingly, whereas TLR responses in innate immune cells from mice expressing kinase-dead IRAK4 were abolished, IRAK4-independent signaling and T-cell receptor signaling appeared intact (19). Likewise, several human cell populations show differing dependences on the kinase activity of IRAK4 for mediating TLR signaling (20–22). However, IRAK4 inhibitors have been the intense focus for therapeutic intervention. Although IRAK4 inhibitors have shown efficacy in murine models of inflammation and autoimmunity, as well as in rare MyD88-driven B-cell lymphomas *in vitro* (23–25), to date, these encouraging results have not been recapitulated in human inflammatory disease.

Despite the critical role myddosome formation plays in initiating TLR-induced signaling, the molecular mechanisms of myddosome formation and subsequent proximal signaling events remain poorly defined. Here, taking both genetic and pharmacological approaches, we show that whereas inhibition of IRAK4 kinase activity blocks TLR cytokine production, the myddosome complex is actually more stable. Importantly, we further found that the kinase activity of IRAK4 is dispensable for TLR-mediated NF-κB signaling but essential for production of inflammatory cytokines. These findings may explain why targeting IRAK4 kinase activity has not been as successful as initially anticipated for treatment of inflammation.

## Results

### Activation of IRAK4 is an early MyD88-dependent TLR signaling event

**Figure 1.**
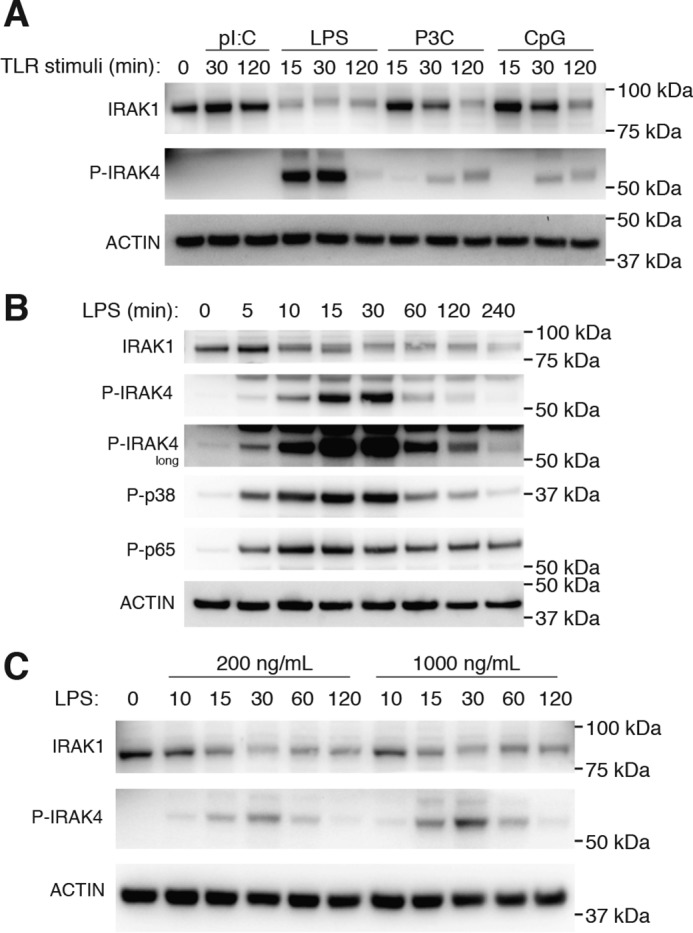
**MyD88-dependent TLR signaling activates IRAK1 and IRAK4.** *A*, BMDMs were left untreated or stimulated with a panel of synthetic TLR ligands for up to 2 h: 10 μg/ml poly(I:C) (pI:C-TLR3); 1 μg/ml LPS (TLR4); 200 ng/ml Pam3CysK4 (P3C-TLR1/2); or 0.1 μm CpG DNA 1826 (TLR9). WCLs were subjected to immunoblotting for IRAK1, P-IRAK4, and actin as a loading control. The data presented are representative of three independent experiments. *B*, BMDMs were left untreated or stimulated with 1 μg/ml LPS from 5 min up to 4 h. WCLs were subjected to immunoblotting for IRAK1, P-IRAK4, P-p38 MAPK, NF-κB P-p65, and actin as a loading control. The data presented are representative of two independent experiments. *C*, BMDMs were left untreated or stimulated with either 200 ng/ml or 1 μg/ml LPS from 10 min up to 2 h. WCLs were subjected to immunoblotting for IRAK1, P-IRAK4, and actin as a loading control. The data presented are representative of three independent experiments.

Loss of the IRAK1 signal by Western blotting is a reliable indicator of early TLR signaling, which depends on the upstream kinase activity of IRAK4 (26). IRAK4 activation occurs via phosphorylation of Thr-345, Ser-346, and Thr-342 within its activation loop, with Thr-345 representing the prototypical residue required for activation (9, 27, 28). Hence, using a highly specific antibody to detect phosphorylation of IRAK4 at Thr-345 and Ser-346 (20), we examined phosphorylated (P)-IRAK4, in addition to IRAK1 loss, by immunoblotting following activation of a panel of TLR agonists in bone marrow–derived macrophages (BMDMs). Activation of TLR4 (with LPS), TLR1/2 (with Pam3CysK4; P3C), or TLR9 (with CpG DNA), all of which require the adaptor MyD88 to engage downstream signaling, led to the appearance of P-IRAK4, with LPS activation resulting in both the fastest kinetics and greatest levels of IRAK4 activation (Fig. 1*A*). In contrast, a distinct decrease in the protein signal of IRAK1 was observed following stimulation of these TLRs (Fig. 1*A*). As a control, cells were also stimulated with poly(I:C) to activate TLR3, which does not require MyD88-IRAK4 to elicit downstream signaling, and as expected did not result in IRAK4 phosphorylation or the loss of IRAK1 (Fig. 1*A*). We next examined the kinetics of IRAK1 loss and P-IRAK4 in greater detail by performing a time course following LPS stimulation. This revealed that LPS-induced phosphorylation of IRAK4 occurred from 5 to 120 min and most robustly between 15 and 30 min, whereas loss of IRAK1 was apparent from 10 min and continued to decrease for up to 4 h (Fig. 1*B*). Phosphorylation of the subsequent signaling molecules, p38 MAPK and NF-κB p65, appeared to closely match the kinetics of P-IRAK4 (Fig. 1*B*). Whereas the magnitude of IRAK4 phosphorylation depended on the dose of LPS, its kinetics did not (Fig. 1*C*). These data demonstrate that phosphorylation of IRAK4 is a specific and early MyD88-dependent event following TLR activation.

### Rapid myddosome formation precedes gradual disassembly

**Figure 2.**
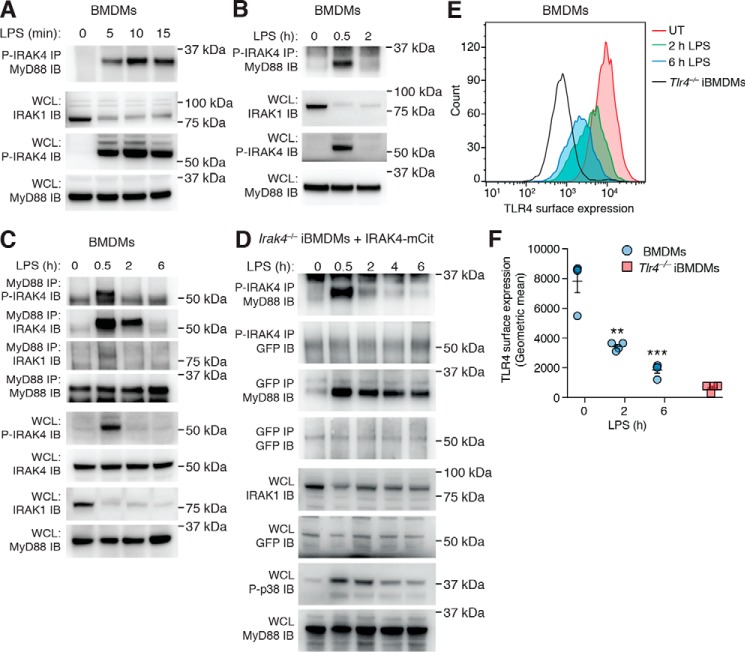
**Myddosome complexes form rapidly but are stable following the loss of P-IRAK4.** BMDMs were left untreated or stimulated with 1 μg/ml LPS for 5, 10, and 15 min (*A*) or 30 min and 2 h (*B*). *A* and *B*, WCLs were then subjected to P-IRAK4 IP followed by MyD88 immunoblotting or directly subjected to immunoblotting for IRAK-1, P-IRAK4, and MyD88, which also served as a loading control. The data presented are representative of three independent experiments. *C*, BMDMs were left untreated or stimulated with 1 μg/ml LPS for 30 min, 2 h, or 6 h. WCLs were then subjected to MyD88 IP, followed by immunoblotting for P-IRAK4, IRAK4, IRAK1, and MyD88, or directly subjected to immunoblotting with the same antibodies. The data presented are representative of three independent experiments. *D*, *Irak4*^−/−^ iBMDMs expressing IRAK4-mCitrine were generated as described under “Experimental procedures.” These cells were left untreated or stimulated with 1 μg/ml LPS for 30 min, 2 h, 4 h, or 6 h before WCLs were divided evenly and subjected to either P-IRAK4 IP or GFP IP followed by immunoblotting for MyD88 and GFP. WCLs were also directly subjected to immunoblotting for IRAK1, GFP, P-p38 MAPK, and MyD88. The data presented are representative of two independent experiments. *E*, BMDMs were left untreated (*UT*) or stimulated with 1 μg/ml LPS for 2 or 6 h before staining with an antibody against the extracellular region of TLR4 and subjected to flow cytometric analysis to determine the level of TLR4 surface expression. *Tlr4*^−/−^ iBMDMs were also stained as a control. One representative experiment is presented. *F*, TLR4 surface expression was then quantified by examining the geometric mean of stained cells. Data are presented as the mean ± S.E. (*error bars*) (untreated *versus* LPS: **, *p* < 0.01; ***, *p* < 0.001) combined from four individual experiments.

The precise details surrounding myddosome formation and disassembly remain elusive. We examined the kinetics of myddosome assembly by immunoprecipitation (IP) experiments, initially focusing on interactions between P-IRAK4 and the adaptor MyD88 following acute LPS stimulation. Performing P-IRAK4 IPs, we observed interactions with MyD88 as early as 5 min following LPS stimulation (Fig. 2*A*). However, examining longer time points, we found that whereas this interaction remained stable at 30 min, by 2 h, it was no longer visible (Fig. 2*B*), which is most likely due to the loss of IRAK4 phosphorylation, as observed in whole-cell lysates (WCLs) (Figs. 1 (*A* and *B*) and 2 (*B* and *C*)). We next performed reciprocal MyD88 IPs and examined interactions with both P-IRAK4 and total IRAK4. In line with Fig. 2*B*, P-IRAK4 interactions with MyD88 were visible at 30 min following LPS stimulation but lost by 2 h (Fig. 2*C*). A weak interaction with IRAK1 was also observed at 30 min post-LPS treatment. Interestingly, the interaction between MyD88 and total IRAK4 remained detectable for at least 2 h following LPS but was markedly reduced by 6 h (Fig. 2*C*).

To examine this further, we generated IRAK4 reporter macrophages. Following production of retroviruses in HEK293T cells (Fig. S1*A*), we reconstituted *Irak4*^−/−^ immortalized BMDMs (iBMDMs) with WT IRAK4 fused to the fluorescent protein mCitrine (mCit) (Fig. 1*B*). This allowed us to perform specific IPs using the mCitrine tag (reactive to GFP antibodies) to examine interactions of total IRAK4 in the myddosome complex. To this end, we stimulated WT IRAK4-mCit reporter macrophages for up to 6 h with LPS, divided the resulting whole-cell lysates, and performed simultaneous IPs for both P-IRAK4 and total IRAK4 (via GFP). Consistent with the findings from BMDMs (Fig. 2, *B* and *C*), P-IRAK4 interactions with MyD88 were markedly reduced by 2 h, whereas the interactions between MyD88 and total IRAK4-mCit appeared stable for up to 6 h following LPS stimulation (Fig. 2*D*).

Upon activation by LPS, TLR4 undergoes CD14-dependent endocytosis to initiate the TRIF signaling pathway (29, 30). Consistent with these reports, we also observed down-modulation of surface TLR4 following LPS stimulation as determined by flow cytometry (Fig. 2, *E* and *F*). These results indicate that the loss of IRAK4 kinase activity is an active process that does not require disassembly of the myddosome, which remains stable over a long period even following internalization of TLR4.

### IRAK4 kinase activity is essential for MyD88-dependent TLR cytokines

**Figure 3.**
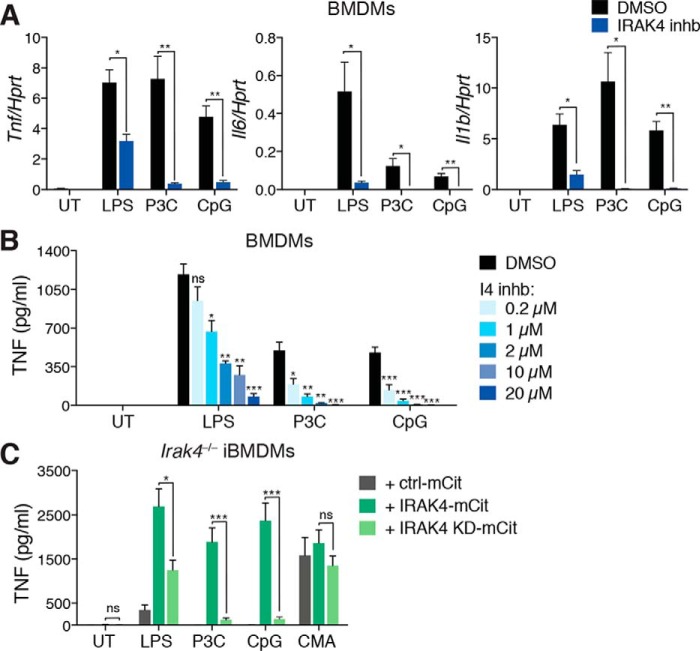
**IRAK4 kinase activity is critical for MyD88-dependent cytokine production.** *A*, BMDMs were pretreated with either DMSO or a 20 μm concentration of a selective IRAK4 inhibitor (I4 inhb) for 30 min. Cells were then left untreated (*UT*) or stimulated with either 1 μg/ml LPS, 500 ng/ml P3C, or 0.5 μm CpG DNA 1826 for a further 4 h. Cells were then lysed for RNA isolation and mRNA analysis by qPCR. Data are presented as the mean ± S.E. (*error bars*) (DMSO *versus* I4 inhb: *, *p* < 0.05; **, *p* < 0.01) combined from three independent experiments. *B*, BMDMs were pretreated with either DMSO or 0.2–20 μm I4 inhb for 30 min. Cells were then left untreated (*UT*) or stimulated with either 1 μg/ml LPS, 500 ng/ml P3C, or 0.5 μm CpG DNA 1826 for a further 4 h. Supernatants were then collected and subjected to ELISA to determine the levels of secreted TNF. Data are presented as the mean ± S.E. (DMSO *versus* I4 inhb: *ns*, not significant; *, *p* < 0.05; **, *p* < 0.01; ***, *p* < 0.001) combined from four individual experiments. *C*, *Irak4*^−/−^ iBMDMs expressing either mCitrine alone, IRAK4-mCitrine, or IRAK4 KD-mCitrine were stimulated with 1 μg/ml LPS, 500 ng/ml P3C, 0.5 μm CpG DNA 1826, or 500 μg/ml CMA (a murine STING ligand) for 4 h. Supernatants were then collected and subjected to ELISA to determine the levels of secreted TNF. Data are presented as the mean ± S.E. (IRAK4-mCit *versus* IRAK4 KD-mCit: *ns*, not significant; *, *p* < 0.05; ***, *p* < 0.001) combined from three individual experiments.

We next examined the effects of both pharmacological and genetic inhibition of IRAK4 kinase activity on TLR-induced cytokine mRNA expression and protein secretion from murine macrophages. First, primary BMDMs were pretreated for 30 min with a potent and highly selective IRAK4 inhibitor or with DMSO alone as a control, before stimulation with several TLR ligands eliciting MyD88-dependent signals for 4 h. IRAK4 kinase inhibition led to significant reductions in TLR-induced *Tnf*, *Il6*, and *Il1b* mRNA expression (Fig. 3*A*), as well as dose-dependent reductions in the secretion of TNF protein, with the most pronounced decreases seen in response to stimulation of TLR1/2 or TLR9 (Fig. 3*B*).

The genetic inhibition of IRAK4 kinase activity was tested by comparing cytokine responses of *Irak4*^−/−^ iBMDMs reconstituted with mCit alone, WT IRAK4-mCit, or a kinase-dead mutant form (K213A/K214A) of IRAK4 (IRAK4 KD) (see Fig. S1). As in the case of pharmacological IRAK4 kinase inhibition, macrophages expressing IRAK4 KD displayed significantly decreased secretion of TNF following 4 h TLR stimulation compared with the WT IRAK4 counterparts, whereas *Irak4*^−/−^ iBMDMs expressing mCit alone were mostly unresponsive (Fig. 3*C*). As a control, we showed that the reporters produce similar levels of TNF via activation of a MyD88-independent pathway using the murine STING ligand, 10-carboxymethyl-9-acridanone (CMA), demonstrating no intrinsic defects in the different cell populations (Fig. 3*C*). It should be noted that the levels of TNF secreted by the IRAK4-mCit iBMDMs were greater than those from primary BMDMs, most likely due to higher IRAK4-mCit expression levels than those seen for endogenous IRAK4 (see Fig. S1). In both cases, cells stimulated with LPS only showed a reduced dependence on IRAK4 kinase activity, which is attributed to TLR4-mediated initiation of the TRIF signaling pathway in addition to those dependent on MyD88 and IRAK4. These data demonstrate that the kinase activity of IRAK4 is critical for MyD88-dependent TLR-induced cytokine mRNA and protein production in mouse macrophages.

### Inhibition of IRAK4 kinase activity stabilizes myddosome complexes

**Figure 4.**
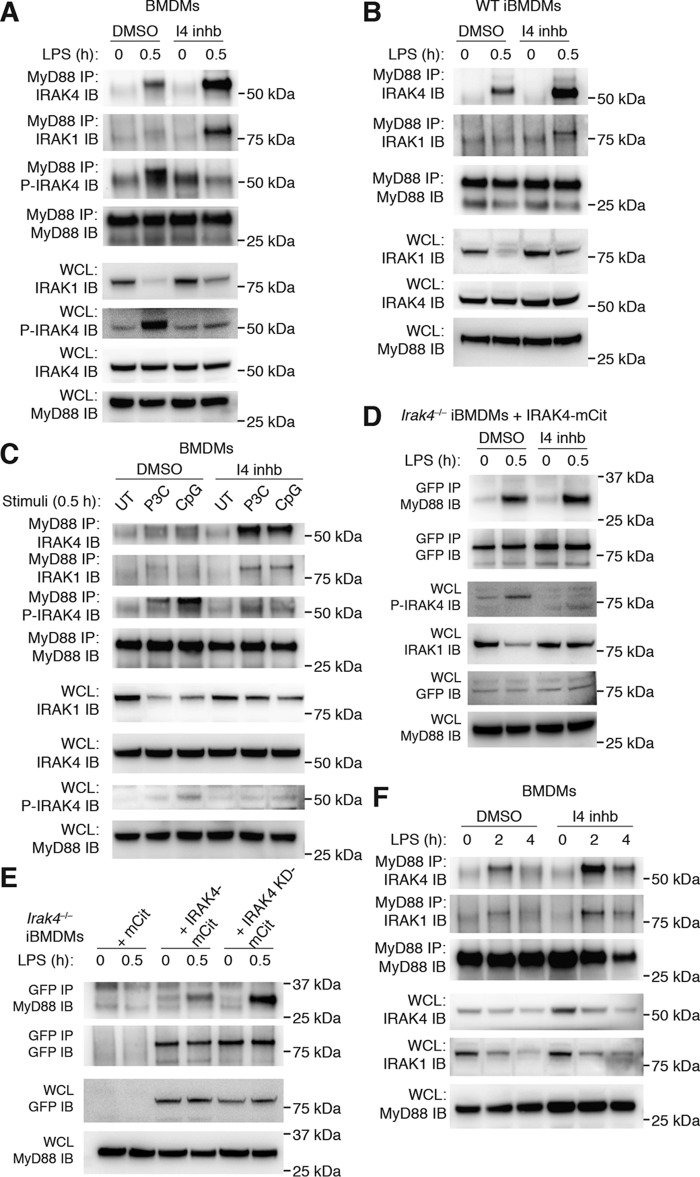
**IRAK4 kinase inhibition stabilizes TLR-induced myddosome interactions.** *A*, BMDMs were pretreated with either DMSO or 20 μm I4 inhb for 30 min. Cells were then left untreated or stimulated with 1 μg/ml LPS for a further 30 min. WCLs were then subjected to MyD88 IP followed by immunoblotting for IRAK4, IRAK1, P-IRAK4, and MyD88 or directly subjected to immunoblotting with the same antibodies. MyD88 immunoblotting also acted as a loading control. The data presented are representative of three independent experiments. *B*, WT iBMDMs were treated as in *A*, before WCLs were subjected to MyD88 IP followed by immunoblotting for IRAK4, IRAK1, and MyD88 or directly subjected to immunoblotting with the same antibodies. The data presented are representative of three independent experiments. *C*, BMDMs were pretreated with either DMSO or 20 μm I4 inhb for 30 min. Cells were then left untreated or stimulated with either 500 ng/ml P3C or 0.5 μm CpG DNA 1826 for a further 30 min. WCLs were then subjected to MyD88 IP followed by immunoblotting for IRAK4, IRAK1, P-IRAK4, and MyD88 or directly subjected to immunoblotting with the same antibodies. MyD88 immunoblotting also acted as a loading control. The data presented are representative of three independent experiments. *D*, *Irak4*^−/−^ iBMDMs expressing IRAK4-mCitrine were treated as in *A* before WCLs were subjected to GFP IP, followed by immunoblotting for MyD88 and GFP, or directly subjected to immunoblotting for P-IRAK4, IRAK1, GFP, and MyD88. The data presented are representative of three independent experiments. *E*, *Irak4*^−/−^ iBMDMs expressing either mCitrine alone, IRAK4-mCitrine, or IRAK4 KD-mCitrine were stimulated with 1 μg/ml LPS for 30 min before WCLs were subjected to GFP IP followed by immunoblotting for MyD88 and GFP or directly subjected to immunoblotting with the same antibodies. The data presented are representative of four independent experiments. *F*, BMDMs were pretreated with either DMSO or 20 μm I4 inhb for 30 min. Cells were then left untreated or stimulated with 1 μg/ml LPS for a further 2 or 4 h. WCLs were then subjected to MyD88 IP, followed by immunoblotting for IRAK4, IRAK1, P-IRAK4, and MyD88, or directly subjected to immunoblotting with the same antibodies. MyD88 immunoblotting also acted as a loading control. The data presented are representative of three independent experiments.

We found that treatment of BMDMs with a specific IRAK4 kinase inhibitor significantly inhibited TLR-induced cytokines (Fig. 3, *A* and *B*). However, our finding that myddosome interactions are stable following dephosphorylation of IRAK4 (Fig. 2, *C* and *D*), together with published observations that IRAK4 kinase activity is not required for interactions with MyD88, IRAK1, or IRAK2 (19, 31), strongly suggested that the kinase activity of IRAK4 is dispensable for myddosome formation. Therefore, we specifically examined the effects of IRAK4 kinase inhibition on the stability of the myddosome complex. In whole-cell lysates, we noted that pretreatment with the IRAK4 inhibitor blocked detectable LPS-induced P-IRAK4 and reduced the decrease of IRAK1 levels, demonstrating efficacy of the inhibitor (Fig. 4*A*). Surprisingly, however, we found that pretreatment with the IRAK4 kinase inhibitor led to a robust increase in the interactions between MyD88 and IRAK4 following LPS stimulation in both primary BMDMs and WT iBMDMs when examined by MyD88 IP (Fig. 4, *A* and *B*). We further interrogated the effect of inhibiting IRAK4 kinase activity on myddosomes formed downstream of other TLRs. Similar to TLR4-induced responses, interactions between MyD88 and IRAK4 were greatly increased in response to TLR1/2 or TLR9 activation in BMDMs pretreated with the IRAK4 inhibitor (Fig. 4*C*). Aside from MyD88 and IRAK4, the proposed myddosome complex also comprises IRAK1 (and/or IRAK2) (6). Interestingly, under conditions of IRAK4 kinase inhibition, we also observed IRAK1 interaction with MyD88, which was almost undetectable in macrophages pretreated with DMSO (Fig. 4, *A–C*). This may be explained by the increased levels of IRAK1 detectable in WCLs following IRAK4 kinase inhibition.

To complement these results, *Irak4*^−/−^ iBMDMs expressing IRAK4-mCit were tested under the same experimental conditions followed by GFP IP, which similarly led to increased complex formation between MyD88 and IRAK4-mCit, albeit less than in WT macrophages (Fig. 4*D*). Finally, we examined the effect of genetic inhibition of IRAK4 kinase activity on myddosome formation using the IRAK4 reporter macrophages generated (Fig. S1). In this case, *Irak4*^−/−^ iBMDMs reconstituted with either mCit alone, IRAK4-mCit, or IRAK KD-mCit were treated for 30 min with LPS and subjected to GFP IP. This approach demonstrated that, like chemical inhibition of IRAK4, genetic ablation of IRAK4 kinase activity also results in increased levels of MyD88 complexed with IRAK4 (Fig. 4*E*). As expected, the *Irak4*^−/−^ control cells expressing mCit alone showed no interaction with MyD88.

We next examined whether inhibition of IRAK4 kinase activity increased the stability of the myddosome over time. To this end, we pretreated BMDMs with DMSO or the IRAK4 inhibitor and examined myddosome interactions 2 and 4 h following LPS stimulation. Similar to Fig. 2*C*, in DMSO pretreated cells, LPS stimulation induced the interaction of MyD88 and IRAK4 at 2 h, which was all but lost by 4 h. In contrast, cells pretreated with the IRAK4 inhibitor showed increased interaction at 2 h and continued association at 4 h (Fig. 4*F*). Together, these data demonstrate that in the absence of IRAK4 kinase activity, MyD88 interacts more strongly with IRAK4 and for longer, thus constituting a considerably more stable myddosome complex. This strongly suggests that IRAK4 plays a significant scaffold function in TLR signaling, independent of its kinase activity.

**Figure 5.**
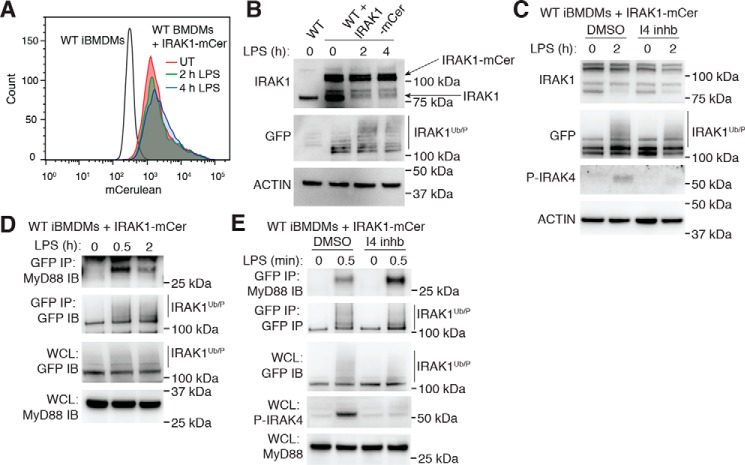
**Examination of IRAK1 in myddosome interactions.** *A* and *B*, WT iBMDMs expressing IRAK1-mCerulean were left untreated (*UT*) or stimulated with 1 μg/ml LPS for 2 or 4 h. *A*, cells were collected and subjected to flow cytometric analysis to determine levels of mCerulean fluorescence. WT iBMDMs alone were also included as a negative control. Data are representative of three independent experiments. *B*, WCLs were subjected to immunoblotting for IRAK1, GFP, and actin as a loading control. Endogenous IRAK1 and IRAK1-mCer are indicated with *arrows*, whereas smears in GFP immunoblots representing ubiquitinated and phosphorylated IRAK1 are indicated with a *line*. Data presented are representative of three independent experiments. *C*, WT iBMDMs expressing IRAK1-mCerulean were pretreated with either DMSO or 20 μm I4 inhb for 30 min. Cells were then left untreated or stimulated with 1 μg/ml LPS for 2 h. WCLs were subjected to immunoblotting for IRAK1, GFP, P-IRAK4, and actin as a loading control. Data presented are representative of three independent experiments. *D*, WT iBMDMs expressing IRAK1-mCerulean were left untreated or stimulated with 1 μg/ml LPS for 30 min or 2 h. WCLs were then subjected to GFP IP followed by immunoblotting for MyD88 and GFP or directly subjected to immunoblotting with the same antibodies. MyD88 in WCLs also served as a loading control. The data presented are representative of three independent experiments. *E*, WT iBMDMs expressing IRAK1-mCerulean were pretreated with either DMSO or 20 μm I4 inhb for 30 min. Cells were then left untreated or stimulated with 1 μg/ml LPS for a further 30 min. WCLs were then subjected to GFP IP followed by immunoblotting with MyD88 and GFP or directly subjected to immunoblotting for GFP, P-IRAK4, and MyD88. The data presented are representative of four independent experiments.

The rapid loss of IRAK1 following TLR activation was initially attributed to proteasomal degradation (32–34). However, recent studies have shown that following activation by IRAK4, IRAK1 actually undergoes significant phosphorylation and ubiquitination events resulting in slower-migrating protein species that are not reactive with most IRAK1 antibodies unless protein lysates are subjected to phosphatase and deubiquitinase treatment (35, 36). As we were only able to clearly observe IRAK1 complexed within the myddosome following treatment of cells with the IRAK4 inhibitor, we generated a reporter cell system in which we could examine activated IRAK1 expression by immunoblotting. To do this, we stably expressed IRAK1 fused to the fluorescent protein, mCerulean (mCer), in WT iBMDMs, enabling us to monitor the signal of both the endogenous IRAK1 and IRAK1-mCer (reactive to GFP antibodies). We treated these IRAK1 reporter cells with LPS for up to 4 h and observed no loss of the mCer signal as examined by flow cytometry (Fig. 5*A*), suggesting that IRAK1-mCer was not degraded within this time frame. Using an antibody against IRAK1, we next examined the effects of LPS treatment on the IRAK1 reporter cells, finding that whereas the signal for endogenous IRAK1 rapidly disappeared after 30 min, the larger IRAK1-mCer species remained detectable up to 4 h post-stimulation (Fig. 5*B*). Probing with a GFP antibody confirmed that IRAK1-mCer is detectable over time and undergoes post-translational modifications, as observed by significant smearing consistent with the altered molecular weight following LPS treatment (Fig. 5*B*). To test that this smearing indeed represents IRAK1 activation, we pretreated the IRAK1 reporter cells with the IRAK4 inhibitor or DMSO before stimulating the cells with LPS. In the presence of the IRAK4 inhibitor, the disappearance of the endogenous IRAK1 signal was reduced, demonstrating efficacy of the inhibitor to prevent IRAK1 activation, whereas the smearing seen in the GFP immunoblot was significantly decreased, demonstrating that IRAK4 induces IRAK1 modifications (Fig. 5*C*). We then examined myddosome formation in the IRAK1-mCer reporter macrophages and found that following GFP IP, we could observe interactions of MyD88 with IRAK1-mCer (Fig. 5*D*). Finally, we examined the effects of IRAK4 kinase inhibition on interactions of IRAK1-mCer and MyD88. Consistent with our previous results, we observed that pretreatment of cells with the IRAK4 inhibitor resulted in increased binding of MyD88 to IRAK1-mCer following LPS stimulation (Fig. 5*E*). These data further demonstrate increased stabilization of the myddosome complex in the absence of IRAK4 kinase activity by increasing the association of IRAK1 with MyD88.

### IRAK4 kinase activity is dispensable for NF-κB and MAPK activation downstream of TLR activation

**Figure 6.**
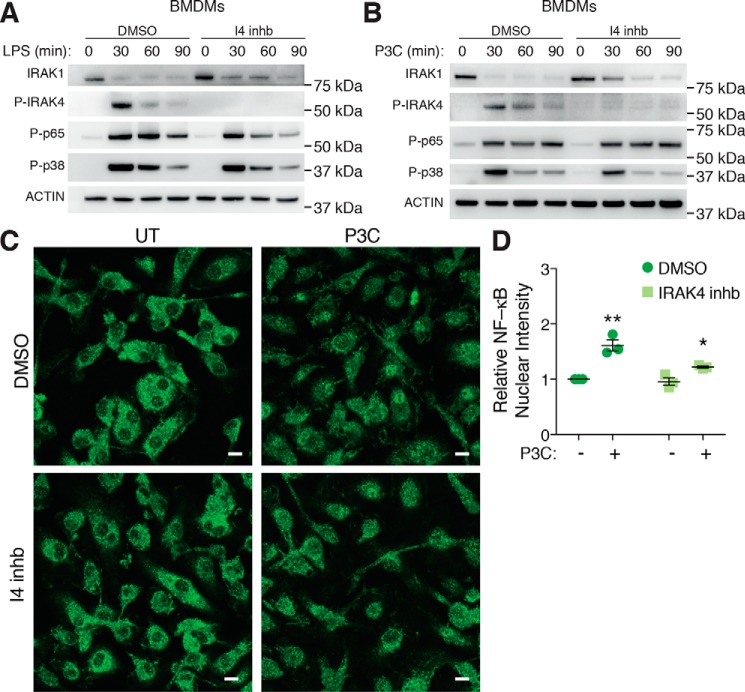
**IRAK4 kinase activity is dispensable for NF-κB and MAPK activation downstream of TLRs.** *A–C*, BMDMs were pretreated with either DMSO or a 20 μm concentration of a selective I4 inhb for 30 min. Cells were then left untreated or stimulated with 1 μg/ml LPS (*A*) or 500 ng/ml P3C (*B*) for an additional 30, 60, or 90 min. WCLs were then subjected to immunoblotting for IRAK1, P-IRAK4, NF-κB P-p65, P-p38 MAPK, and actin as a loading control. Data presented are representative of at least three independent experiments. *C*, cells were then left untreated (*UT*) or stimulated with 500 ng/ml P3C for an additional 4 h before fixation and staining with an NF-κB P-p65 antibody, followed by a specific secondary antibody conjugated to an Alexa-488 fluorophore. Samples were then examined by confocal microscopy. Representative images are presented from three biological replicates. *Scale bar*, 10 μm. *D*, NF-κB P-p65 nuclear translocation was then quantified by examining the mean fluorescence intensity of the Alexa-488 signal in cell nuclei. Nuclear intensity was made relative to DMSO-untreated samples and presented as the mean ± S.E. (*error bars*) (DMSO *versus* IRAK4 inhb: *, *p* < 0.05; **, *p* < 0.01) combined from BMDMs derived from three individual mice (biological replicates).

We have found that inhibition of IRAK4 can strongly inhibit MyD88-dependent TLR cytokine production (Fig. 3) while unexpectedly stabilizing the myddosome complex (Figs. 4 and 5). To further identify the mechanisms underlying ablation of TLR cytokine responses by IRAK4 kinase inhibition, we examined its effects on signaling molecules downstream of TLR activation. First, we pretreated BMDMs with DMSO or the IRAK4 inhibitor before stimulating cells for 30–90 min with LPS to elicit TLR4 activation. This revealed that although BMDMs pretreated with the IRAK4 inhibitor showed a loss of P-IRAK4, only a minor effect was seen on IRAK1 levels and the activation of both NF-κB p65 and p38 MAPK (Fig. 6*A*). We next examined TLR1/2, an entirely MyD88-dependent pathway to account for any compensatory effects of TRIF signaling during TLR4 responses. Again, whereas P-IRAK4 was abolished, we only observed minor effects on the other TLR signaling molecules examined (Fig. 6*B*).

We then examined the nuclear translocation of NF-κB p65 following IRAK4 kinase inhibition. Similar to the phosphorylation of NF-κB p65 (Fig. 6*B*), IRAK4 kinase inhibition modestly reduced TLR1/2-induced NF-κB p65 translocation into the nucleus; however, this response was not totally ablated (Fig. 6, *C* and *D*). These results demonstrate that specific inhibition of IRAK4 activity has minimal effects on the activation of NF-κB and MAPK signaling, as well as NF-κB p65 nuclear translocation downstream of TLR activation. Importantly, these findings are supported by recent work demonstrating that the kinetics of NF-κB translocation are a critical determinant for levels of TNF cytokine production (13).

## Discussion

The molecular integrity of the myddosome is critically required to facilitate downstream TLR signal transduction. However, to date, the molecular mechanisms controlling assembly, stability, kinetics, and composition of myddosome signaling complexes remain poorly understood. Here, we isolated myddosome complexes from TLR-activated primary mouse macrophages and from IRAK reporter macrophages, demonstrating rapid assembly and slow disassembly kinetics. To our knowledge, this is the first full examination of the kinetics of the myddosome.

IRAK4 is thought to become activated via *trans*-autophosphorylation following incorporation into the myddosome structure (9). Hence, our novel approach of isolating myddosome complexes in primary mouse macrophages via P-IRAK4 IP represents interactions within the active structure, whereas reciprocal IPs of MyD88 allowed us to examine the total constituents of the myddosome, even in the absence of P-IRAK4. This was further explored via generation of IRAK4-mCit and IRAK1-mCer reporter macrophages to examine interaction with total IRAK4 or IRAK1, respectively, via GFP IPs. Interestingly, these approaches led us to observe that whereas P-IRAK4 appears to be lost around 30 min post-LPS stimulation, the interactions of total IRAK4, MyD88, and IRAK1 are stable for up to 2 h. Collectively, these data show that IRAK4 kinase function is actively shut off within the myddosome complex. Additionally, this demonstrates that IRAK4 dephosphorylation appears to occur independent of myddosome disassembly and is therefore unlikely to initiate this process. Together with our findings that IRAK4 inhibition or expression of a kinase-dead IRAK4 failed to inhibit myddosome formation, these data suggest that the catalytic activity of IRAK4 is dispensable for both assembly and disassembly of the myddosome.

Utilizing single-molecule fluorescence microscopy of GFP-tagged MyD88 in *Myd88*^−/−^ iBMDMs, Bryant and colleagues showed that MyD88 rapidly forms membrane-bound macromolecular complexes, which are removed from the cell surface within about 10 min following LPS stimulation (13). LPS stimulation did not induce formation of TLR4 oligomers on the cell surface, suggesting a stoichiometric mismatch between activated receptor complexes and oligomeric myddosomes (13). These observations from live single cells, together with our findings that stable myddosome interactions can be detected for up to 2 h from LPS-stimulated BMDMs using biochemical analysis of whole cellular lysates, suggest that following an initial TIR-driven process mediated by activation of surface TLR4–MD-2 receptor complexes, myddosomes may detach from the intracellular domains of TLR4 and be released into the cytosol. This idea is strengthened by observations that a specific *de novo* mutation in the TIR domain of MyD88 (L265P) leads to constitutive myddosome complexes driving aberrant NF-κB activation and inflammation in a number of B-cell human lymphomas (23), which to date has not been shown to be dependent on TIR domains from TLRs.

Surprisingly, we found that a loss of IRAK4 activity by either chemical inhibition or genetic manipulation resulted in a significantly more stable myddosome structure. This increase in stability is suggestive of a prominent protein scaffold role of IRAK4, independent of its kinase activity, in which IRAK4 interacts with MyD88 and IRAK1, tethering them together into the myddosome complex. Indeed, several earlier reports noted higher-affinity interactions of MyD88, TRAF6, and IRAKs with kinase-inactive versions of IRAK1 or IRAK4 in overexpression studies (26, 31, 37, 38). Additionally, using luminescence-based mammalian interactome mapping (LUMIER), a technique enabling examination of protein–protein interactions (39), it was shown that the association of MyD88 with IRAK4 KD is significantly greater (∼5-fold) compared with that of WT IRAK4 (40). The phosphorylation of IRAK proteins may reduce intrinsic protein–protein binding affinities within the myddosome by facilitating changes in surface-binding interfaces within their respective kinase domains or changes in their overall protein conformation. This is supported by the observation that dephosphorylation of full-length IRAK4 in solution with λ-phosphatase leads to formation of stable dimeric complexes, whereas the addition of ATP induces autophosphorylated monomeric IRAK4 (9). Furthermore, the same was true when the kinase domain of IRAK4 alone was examined (9). This is highly suggestive that the kinase domain of IRAK4 plays a critical role in stabilizing IRAK4 dimers and likely the myddosome complex itself, independent of the DD–DD interactions that organize the primary complex formation.

**Figure 7.**
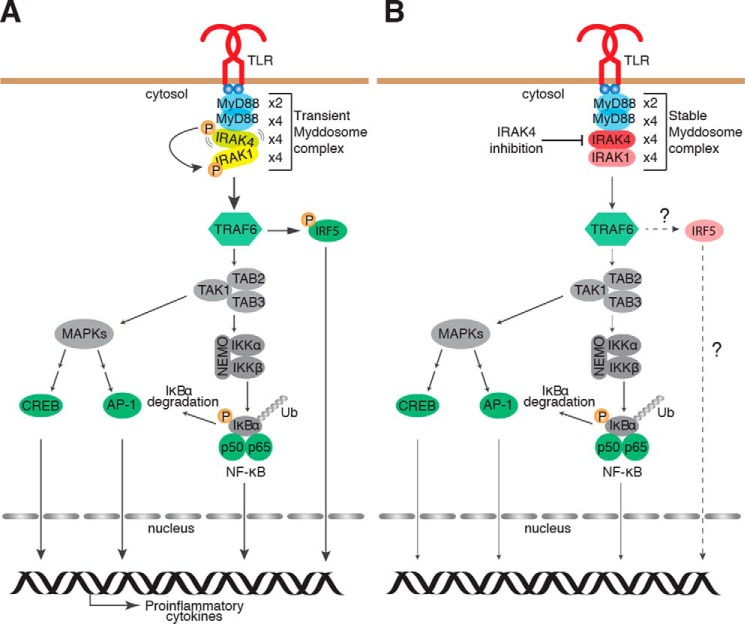
**A model of MyD88-dependent TLR signaling events in the presence and absence of IRAK4 kinase activity.** *A*, activation of TLR dimers at membranes leads to rapid recruitment of the TIR adaptor MyD88 and the ordered assembly of an oligomeric signaling platform termed the myddosome, which comprises four layers: 2× molecules of MyD88, 4× molecules of MyD88, 4× molecules of IRAK4, and 4× molecules of IRAK1/2. Myddosome formation facilitates IRAK4 autophosphorylation and subsequent phosphorylation of IRAK1. The E3 Ub ligase, TRAF6, is then thought to simultaneously activate signaling pathways leading to the transcriptional activity of NF-κB, AP-1 and cAMP-response element–binding protein (via the MAPK pathway), and IRF5, which act in concert for the production of inflammatory cytokines. *B*, in the absence of IRAK4 kinase activity, the myddosome assembles into a more stable complex following TLR activation. The activation of NF-κB and MAPK signaling is only moderately affected by IRAK4 inhibition.

We identified that the kinase activity of IRAK4 is critical for MyD88-dependent cytokine production but is unnecessary for myddosome formation, activation and nuclear translocation of NF-κB p65, and activation of the MAPK pathway. Interestingly, like IRAK4 kinase activity, the enzymatic function of TRAF6 was shown to be essential for cytokine production but play only a minor role in the activation of NF-κB and MAPK signaling molecules following TLR activation (41). These paradoxical observations are made clearer by recent findings from human monocytes, where the kinase activity of IRAK4 was critical for TLR7/8 cytokine responses via specific control of nuclear translocation of IRF5 but redundant for NF-κB (42). These data, together with our observation, strongly support a model in which IRAK4 plays a dual role in myddosome formation and TLR signaling in macrophages. Primarily, IRAK4 itself is essential as a scaffold molecule during the assembly of the myddosome, facilitating NF-κB and MAPK signaling (Fig. 7*A*). Although dispensable for NF-κB activation, the kinase activity of IRAK4 is specifically required to elicit an inflammatory cytokine response (Fig. 7*B*), which may be mediated via activation of IRAK1/2 and the E3 Ub ligase activity of TRAF6.

For some time, IRAK4 kinase inhibitors have been an attractive target for therapeutic use in chronic inflammatory and autoimmune diseases. Interestingly, IRAK4 kinase inhibition has selective effects on cytokine responses from differing human cell populations (20). Our findings that IRAK4 inhibition maintains the scaffolding function of IRAK4 for myddosome-dependent NF-κB and MAPK activation may help to explain these observations. Although IRAK4 kinase inhibitors have shown great efficacy in various murine inflammatory disease models (24, 43), it is yet to be determined whether this will translate to positive outcomes in human conditions. Our findings may help explain why, to date, targeting IRAK4 kinase activity has not been more successful. Furthermore, we highlight that targeting the scaffold function of IRAK4 may be an attractive alternative for therapeutic inhibition.

## Experimental procedures

### Reagents

Reagents included were as follows: LPS from *Escherichia coli*, serotype EH100 (Ra)-TLR grade (Enzo Life Sciences); P3C, 1826 CpG DNA, high-molecular weight poly(I:C) (Invivogen); DMSO, CMA, also known as 9-oxo-10(9H)-acridineacetic acid (Sigma-Aldrich); IRAK4 inhibitor, also known as PF-06426779 or compound 38 (Pfizer) (44).

### Cell culture

The use of mice in this study for generating primary BMDMs was approved by the Walter and Eliza Hall Institute Animal Ethics Committee. BMDMs were obtained by culturing bone marrow cells harvested from 6–8-week-old WT C57BL/6 mice in Dulbecco′s modified Eagle′s medium (DMEM) supplemented with 10% fetal bovine serum (FBS; Sigma-Aldrich), 100 units/ml penicillin, 100 μg/ml streptomycin, and 20% L929 conditioned medium at 37 °C in a humidified atmosphere with 10% CO_2_. Six days later, adherent BMDMs were harvested and plated for experiments. HEK293T cells and iBMDMs (WT, *Irak4*^−/−^, and *Tlr4*^−/−^) (45) were maintained in DMEM with 10% FBS, 100 units/ml penicillin, and 100 μg/ml streptomycin at 37 °C in a humidified atmosphere with 10% CO_2_.

### Generation of IRAK reporter macrophages

Retroviral plasmids expressing murine IRAK4 (pR-IRAK4-mCitrine) or a KD version of IRAK4 (pR-IRAK4 KD^K213A/K214A^-mCitrine) fused to mCitrine at the C-terminus were generated as described previously (16). The pRH-IRAK1-mCerulean retroviral plasmid expressing murine IRAK1-mCerulean was generated by amplifying murine IRAK1 from the pEF-V5-IRAK1 expression vector (31) using specific primers (forward, 5′-tttggatccATGGCCGGGGGGCCGG-3′; reverse, 5′-aaactcgagGCTCTGGAATTCATCACTTTCTTCAGGTC-3′) before the resultant PCR product was digested with BamHI and XhoI and cloned in-frame into the corresponding sites of the pRH-mCerulean retroviral vector. *Irak4*^−/−^ iBMDMs expressing either mCitrine alone, IRAK4-mCitrine, or IRAK4 KD-mCitrine were generated by retroviral transduction with the pR-mCitrine, pR-IRAK4-mCitrine, or pR-IRAK4 KD^K213A/K214A^-mCitrine plasmids, respectively. Following retroviral transduction, cells were then sorted based on positive expression of mCitrine to approximately the same levels between the three cell lines. WT iBMDMs expressing murine IRAK1-mCerulean were generated by retroviral transduction with the pRH-IRAK1-mCerulean plasmid. In this case, positive cells were selected via antibiotic treatment with 600 μg/ml hygromycin (Invivogen) for at least 7 days. All retroviral transduction and selection was performed using a well-described protocol (46).

### Cytokine mRNA analysis by quantitative PCR

RNA was isolated using the EZ-10 DNAaway RNA Miniprep Kit (Bio Basic) according to the manufacturer's protocol before cDNA synthesis. Quantitative real-time PCR (qPCR) was performed on cDNA using the Maxima SYBR Green/ROX qPCR Master Mix (Thermo Scientific) on a Viia 7 real-time PCR system (Thermo Scientific). Primer sequences used were as described previously (47). Expression of target genes was normalized to respective housekeeping genes. Cytokine mRNA expression data are presented as mean ± S.E., from three combined experiments.

### Measurement of secreted cytokines by ELISA

Levels of murine TNF cytokine in cell culture supernatants were measured by ELISA (Thermo Scientific) as per the manufacturer's instructions. ELISA data are presented as mean ± S.E., from at least three combined experiments.

### Generation of whole-cell lysates

For immunoprecipitation experiments, ∼10 × 10^6^ iBMDMs or 20 × 10^6^ BMDMs were lysed on ice for 30 min with 750 μl of 1× Nonidet P-40 buffer (1% Nonidet P-40, 20 mm Tris-HCl, pH 7.4, 150 mm NaCl, 1 mm EGTA, 10% glycerol, 10 mm NaPP_i_, 5 mm NaF, and 1 mm Na_3_VO_4_) supplemented with 1 mm phenylmethylsulfonyl fluoride and cOmplete protease inhibitors (Roche Applied Science). For immunoblotting experiments, ∼1.0 × 10^6^ iBMDMs or BMDMs were lysed on ice with 120 μl of 1× radioimmune precipitation buffer (20 mm Tris-HCl, pH 7.4, 150 mm NaCl, 1 mm EDTA, 1% Triton X-100, 10% glycerol, 0.1% SDS, 0.5% deoxycholate, 10 mm NaPP_i_, 5 mm NaF, and 1 mm Na_3_VO_4_) supplemented with 1 mm phenylmethylsulfonyl fluoride and cOmplete protease inhibitors (Roche Applied Science). Whole-cell lysates were subsequently clarified by centrifugation at 13,000 × *g* for 10 min at 4 °C.

### Immunoblotting

For immunoblotting experiments, 60 μl of whole-cell lysate were diluted in 20 μl of 4× reducing SDS-PAGE sample loading buffer (1.25% SDS, 12.5% glycerol, 62.5 mm Tris-HCl, pH 6.8, 0.005% bromphenol blue, 50 mm DTT) and heated to 95 °C for 10 min before 20–35 μl was run on Novex 4–12% precast SDS-polyacrylamide gels (Thermo Scientific) with MES running buffer (Thermo Scientific). Separated proteins were transferred onto polyvinylidene difluoride membranes (Millipore) and blocked in 5% skim milk powder in PBS with Tween 20 before overnight incubation with specific primary antibodies: anti-MyD88 (R&D Systems; AF3109, 1:1000), anti-P-IRAK4 Thr-345/Ser-346 (Pfizer (20), 1:500), anti-IRAK4 (Thermo Scientific; clone 2H9, MA5-15883, 1:500), anti-IRAK1 (Santa Cruz Biotechnology, Inc.; clone H-273, sc-7883, 1:1000 (discontinued)), anti-P-p65 Ser-536 (Cell Signaling Technology; clone 93H1, 3033, 1:500), anti-P-p38 Thr-180/Tyr-182 (Cell Signaling Technology; 9211, 1:1000), anti-GFP (Thermo Scientific; A-11122, 1:1000), or anti-actin (Santa Cruz Biotechnology; clone I-19, sc-1616, 1:2000 (discontinued)). Membranes were then washed and incubated with appropriate secondary antibodies, and immunoreactivity was imaged using the ChemiDoc Touch Imaging System (Bio-Rad).

### Isolation of myddosome complexes by immunoprecipitation

Following preparation of samples for immunoblotting, 1–2 μg of primary antibody was added to the remaining whole-cell lysate: anti-MyD88 (R&D Systems; AF3109), anti-P-IRAK4 Thr-345/Ser-346 (Pfizer (20)), or anti-GFP (Thermo Scientific; clone E36, A-11120). Samples were then incubated at 4 °C for up to 2 h (or overnight) on a rotator before 50 μl of Dynabeads Protein A (Thermo Scientific; 10002D) or Dynabeads Protein G (Thermo Scientific; 10004D) were added. Samples were then incubated once more at 4 °C for up to 2 h on a rotator before beads were extensively washed with lysis buffer using a DynaMag-2 magnet (Thermo Scientific; 12321D), and proteins were eluted by the addition of 35 μl of 2× reducing SDS-PAGE sample loading buffer (2.5% SDS, 25% glycerol, 125 mm Tris-HCl, pH 6.8, 0.01% bromphenol blue, 100 mm DTT) and heating at 95 °C for 10 min. Samples were then subjected to immunoblotting as described above.

### Measuring TLR4 surface expression by flow cytometry

Approximately 1.0 × 10^6^ BMDMs or *Tlr4*^−/−^ iBMDMs were harvested on ice using PBS containing 2% FBS. Nonspecific Fc receptor binding sites were blocked using an anti-mouse CD16/32 antibody (BioLegend; clone 93, 101301, 1:400) before cells were stained with a PE/Cy7 anti-mouse CD284 (TLR4) antibody (BioLegend; clone SA15-21, 145408, 1:100), and samples were analyzed on an LSR Fortessa cell analyzer (BD Biosciences).

### Immunofluorescence (IF) of NF-κB p65 nuclear translocation

Eight-well μ-slides (Ibidi) were coated with poly-l-lysine (Sigma) before BMDMs were seeded at 2 × 10^5^ cells/well and experiments were performed. Cells were then fixed with 4% paraformaldehyde for 30 min, before blocking and permeabilizing cells (IF buffer: PBS, 10% FBS, 0.5% Triton X-100) for 1 h, before staining with a rabbit monoclonal anti-NF-κB p65 (Cell Signaling Technology; clone C22B4, 4764, 1:100) overnight at 4 °C. Cells were then stained with a secondary donkey anti-rabbit Alexa-488 antibody (Thermo Scientific; R37118, 1:1000) for 1 h at room temperature, before nuclear staining with 4′,6-diamidino-2-phenylindole (1 μm) for 5–10 min. After each step, cells were washed 2–3 times with IF buffer or PBS. Cells were imaged using a Zeiss LSM 780 confocal microscope; 2 × 2 tile scans were obtained for each experimental condition using a ×40 oil objective with Immersol 518 F (Zeiss) and acquired with ZEN 2012 version 8.1 software (Zeiss). Images were generated as tagged image bitmap files (TIFF) using FIJI software. Quantification of nuclear NF-κB p65 was performed using FIJI software by overlaying nuclei identified with 4′,6-diamidino-2-phenylindole staining (not shown) onto the Alexa-488 channel before measurement of mean fluorescent intensity within each nucleus.

### Statistical analysis

Analyses were performed with Prism (GraphPad Software, Inc.), and data are typically presented as the mean ± S.E., where a *p* value < 0.05 was considered significant, as determined by an unpaired two-tailed Student's *t* test.

## Author contributions

D. D. N. conceptualization; D. D. N. and V. R. R. resources; D. D. N., K. R. B., and Y. C. G. data curation; D. D. N., K. R. B., Y. C. G., and E. L. formal analysis; D. D. N., E. L., and S. L. M. supervision; D. D. N., E. L., and S. L. M. funding acquisition; D. D. N. validation; D. D. N., K. R. B., and Y. C. G. investigation; D. D. N. visualization; D. D. N., K. R. B., and Y. C. G. methodology; D. D. N. writing-original draft; D. D. N. project administration; D. D. N., K. R. B., Y. C. G., V. R. R., E. L., and S. L. M. writing-review and editing.

## Supplementary Material

Supporting Information

## Footnotes

4 The abbreviations used are:

TLR: Toll-like receptor
TIR: Toll/interleukin-1 receptor
IRAK: interleukin-1 receptor–associated kinase
MyD88: myeloid differentiation primary response 88
NF-κB: nuclear factor κ-light-chain-enhancer of activated B cells
MAPK: mitogen-activated protein kinase
BMDM: bone marrow–derived macrophage
iBMDM: immortalized bone marrow–derived macrophage
IL: interleukin
TRIF: TIR domain–containing adapter–inducing interferon-β
DD: death domain
Ub: ubiquitin
P-: phosphorylated
P3C: Pam3CysK4
IP: immunoprecipitation
LPS: lipopolysaccharide
mCit: mCitrine
KD: kinase-dead
TNF: tumor necrosis factor
WCL: whole-cell lysate
mCer: mCerulean
CMA: 10-carboxymethyl-9-acridanone
FBS: fetal bovine serum
qPCR: quantitative real-time PCR
IF: immunofluorescence
I4 inhb: IRAK4 inhibitor.
