# Supplementary material for: Interleukin-1 receptor–associated kinase 4 (IRAK4) plays a dual role in myddosome formation and Toll-like receptor signaling
Source: J Biol Chem. 2018 Aug 3;293(39):15195–207. doi: 10.1074/jbc.RA118.003314 (PMC6166714; doi:10.1074/jbc.RA118.003314)
Supplement: Supporting Information [file supp_293_39_15195__index.html]

Interleukin 1 receptor–associated kinase 4 (IRAK4) plays a dual role in Myddosome formation and Toll-like receptor signalling — The dual role of IRAK4 in the Myddosome — Interleukin-1 receptor–associated kinase 4 (IRAK4) plays a dual role in myddosome formation and Toll-like receptor signaling — The dual role of IRAK4 in the myddosome — Supporting Information 

# Interleukin-1 receptor–associated kinase 4 (IRAK4) plays a dual role in myddosome formation and Toll-like receptor signaling

## Supporting Information

- Sup Figure 1 and Legend - Supplemental Figure 1 and Legend
