# Supplementary material for: Interleukin-1 receptor–associated kinase 4 (IRAK4) plays a dual role in myddosome formation and Toll-like receptor signaling
Source: J Biol Chem. 2018 Aug 3;293(39):15195–207. doi: 10.1074/jbc.RA118.003314 (PMC6166714; doi:10.1074/jbc.RA118.003314)
Supplement: Supporting Information [file supp_RA118.003314_137233_1_supp_171838_pcbks4.pdf]

S.Figure 1

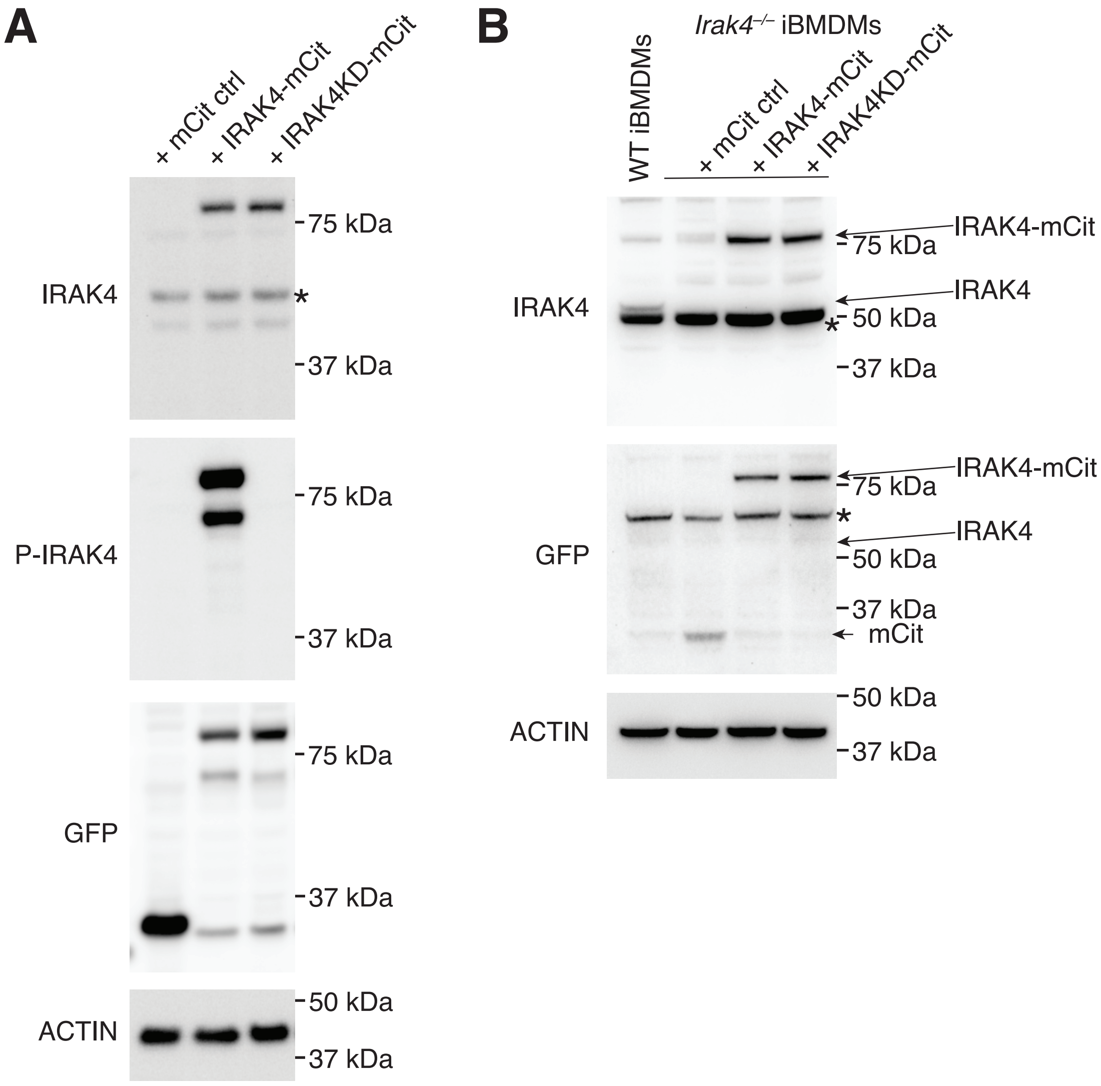

Supporting Information Figure 1. Generation of IRAK4-mCitrine reporter macrophages. (A) HEK293T cells were transiently transfected with plasmids expressing either mCitrine alone, IRAK4-mCitrine or IRAK4 KD-mCitrine. WCLs were then subjected to immunoblot for IRAK4, P-IRAK4, GFP and ACTIN as a loading control. Asterisk indicates a non-specific band. Data is representative of two independent experiments (B) WCLs were generated from WT iBMDMs or *Irak4*$^{-/-}$ iBMDMs stably expressing either mCitrine alone, IRAK4-mCitrine or IRAK4 KD were subjected to immunoblot for IRAK4, GFP or ACTIN as a loading control. Arrows indicate IRAK4-mCit, endogenous IRAK4 and mCit alone while asterisks indicate non-specific bands. Data is representative of three independent experiments.
